# Transient ischemic attack and coronary artery disease: a two-sample Mendelian randomization analysis

**DOI:** 10.3389/fcvm.2023.1192664

**Published:** 2023-08-21

**Authors:** Xiaoyi Qi, Shijia Wang, Liangxian Qiu, Xiongbiao Chen, Qianwen Huang, Kunfu Ouyang, Yanjun Chen

**Affiliations:** ^1^ Departments of Cardiology, Peking University Shenzhen Hospital, Shenzhen, China; ^2^ Medical College, Shantou University, Shantou, China; ^3^ Department of Cardiovascular Surgery, Peking University Shenzhen Hospital, Shenzhen, China

**Keywords:** coronary artery disease, transient ischemic attack, Mendelian randomization, causal relationship, GWAS—genome-wide association study

## Abstract

**Background:**

Although observational studies have shown that patients who experienced transient ischemic attacks (TIAs) had a higher risk of coronary artery disease (CAD), the causal relationship is ambiguous.

**Methods:**

We conducted a two-sample Mendelian randomization (MR) study to analyze the causal relationship between TIA and CAD using data from the FinnGen genome-wide association study. Analysis was performed using the inverse-variance weighted (IVW) method. The robustness of the results was evaluated using MR-Egger regression, the weighted median, MR pleiotropy residual sum, and outlier (MR-PRESSO) and multivariable MR analysis.

**Results:**

Results from IVW random-effect model showed that TIA was associated with an increased risk of coronary artery atherosclerosis (OR 1.17, 95% CI 1.06–1.28, *P* = 0.002), ischemic heart disease (OR 1.15, 95% CI 1.04–1.27, *P* = 0.007), and myocardial infarction (OR1.15, 95% CI 1.02–1.29, *P* = 0.025). In addition, heterogeneity and horizontal pleiotropy were observed in the ischemic heart disease results, while the sensitivity analysis revealed no evidence of horizontal pleiotropy in other outcomes.

**Conclusions:**

This MR study demonstrated a potential causal relationship between TIA and CAD. Further research should be conducted to investigate the mechanism underlying the association.

## Introduction

Coronary artery disease (CAD) is a cardiovascular disorder caused by atherosclerosis or atherosclerotic occlusions of the coronary arteries (1). Coronary artery atherosclerosis (CAA) is a complex and chronic inflammatory disease characterized by atherosclerotic plaque formation of coronary arteries and has various clinical manifestations. CAD includes a series of diseases that belong to different stages of the pathology progression of coronary atherosclerosis. A ruptured plaque with occlusion of the coronary artery results in myocardial infarction (MI). Acute MI and myocardial necrosis could further induce left ventricular dysfunction and ischemic heart disease (IHD) (2). CAD is a leading cause of death in both developed and developing countries (3). According to the Global Burden of Diseases 2016, 17.8 million patients die annually from cardiovascular disease, accounting for 21.1% of death overall global deaths (4). The high morbidity and mortality associated with CAD also impose significant economic burdens (5). Thus, identifying the risk factors of CAD is necessary to prevent and reduce the disease burden.

Transient ischemic attack (TIA) is defined as a sudden, focal neurological deficit of presumed vascular origin that lasts less than 24 h (6). Approximately 240,000 individuals per year in the USA experience TIA (7). Even though TIA results in transient neurological symptoms, its comorbidity such as recurrent stroke and cardiac events should not be ignored. Previous studies have indicated that CAD is a common comorbidity in patients with TIA. An early Oxfordshire community stroke project (OCSP) found that patients with TIA have a 27.8% risk of developing CAD within 10 years (8). A study on the relationship between ischemic stroke and CAD found that stroke patients were five times more likely to have coronary artery plaque (9). A retrospective study showed that the incidence of MI in TIA patients was higher than that in the general population (OR 2.09, 95% CI 1.52–2.81) (10). Furthermore, a meta-analysis including 58 studies found that the annual risk of MI is 1.67% greater in patients with a history of TIA (11). According to the Third China National Stroke Registry (CNSR-III), the 1-year risk of MI or vascular death due to cardiovascular disease is 11.2% in TIA patients (12). However, the prevalence of non-fatal CAD in Japan is much lower, only 1.9% (13). Besides, the results of observational studies could not avoid reverse causality and confounding factors (14). Therefore, further studies are necessary to elucidate whether there is a causal association between TIA and CAD.

Mendelian randomization (MR) is a novel approach to evaluating causal links between risk factors and outcomes. The basis of MR is that genetic variants that affect a specific risk factor are randomly distributed in a population. In addition, it is assumed that the genetic variants are not associated with confounding factors. Consequently, differences in outcomes can be attributed to the differences in risk factors (15). In this study, we conducted a two-sample Mendelian randomization analysis to explore the potential causal relationship between TIA and CAD. The identification of a causal relationship between TIA and CAD could contribute to reducing and preventing cardiac events in patients experiencing TIA.

## Methods

### Data sources

To perform two-sample MR analyses, we obtained genome-wide association study (GWAS) summary statistics from the MR-Base platform (http://gwas-api.mrcieu.ac.uk/). Summary-level data for TIA were obtained from the FinnGen study (16), which includes 8,835 cases and 202,223 controls to date. Data on CAA, IHD, and MI were obtained from the FinnGen study (16), with 23,363 CAA and 187,840 controls, 30,952 IHD cases, with 187,840 controls, and 12,801 MI cases with 187,840 controls. The enrolled participants were all of European ancestry. Apart from the primary instruments, data on confounding factors, such as smoking, diabetes, and hypertension, were also extracted from the FinnGen study (16), with a total of 218,754, 215,654, and 138,088 participants of European ancestry. Table 1 provides detailed information on the GWAS data sources.

**Table 1:** Sources of GWAS data.

Trait	Sample size	Source	GWAS ID
Transient ischemic attack	211,058	Europe	finn-b-I9_TIA
Coronary atherosclerosis	211,203	Europe	finn-b-I9_CORATHER
Myocardial infarction	200,641	Europe	finn-b-I9_MI
Ischemic heart disease	218,792	Europe	finn-b-I9_ISCHHEART
Hypertension	218,754	Europe	finn-b-I9_HYPTENS
Diabetes	215,654	Europe	finn-b-E4_DM2
Smoking	138,088	Europe	finn-b-SMOKING

### MR assumptions and instrumental variable selection

**Figure 1:**
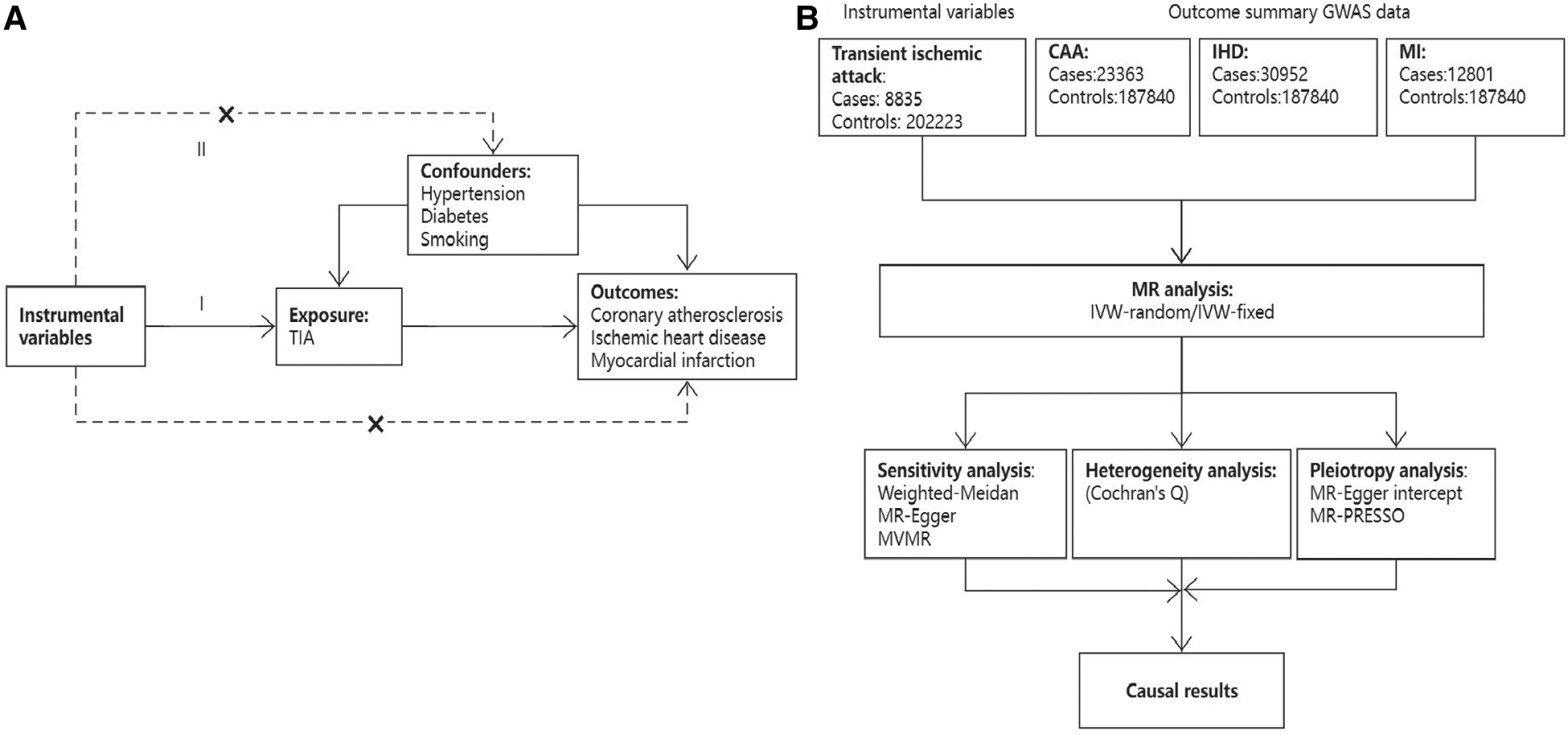
An overview of the present study design. (A) Three basic assumptions of the MR study; (B) Flow chart of study design.

This two-sample Mendelian randomization analysis was conducted in accordance with the latest Strengthening the Reporting of Observational Studies in Epidemiology using Mendelian Randomization (STROBE-MR) guideline (Figure 1) (17). A causal association between TIA and CAD could be inferred if three basic MR assumptions were satisfied: (1) Instrumental variables (IVs) directly affected exposure (relevance assumption); (2) IVs were not associated with other confounders (independence assumption); (3) IVs affected the risks of outcomes through exposure, not through other pathways (exclusivity assumption). Instrumental variables were selected according to the following criteria. First, genetic variants significantly associated with TIA (*P* < 5 × 10^−6^) in a GWAS study were included. Second, SNPs with a threshold linkage disequilibrium (LD) of *r*^2^ > 0.001 were excluded to ensure independence between SNPs. Third, SNPs with an *F*-statistic less than 10 were excluded to avoid weak IV bias. The following equation was used to calculate the *F*-statistic: *F* = *R*^2^(*N*-*K*-1)/[*K*(1-*R*^2^)], where *N* denotes the GWAS sample; *K* refers to the number of SNPs in the MR analysis; and *R*^2^ is the cumulative explained variance of the selected SNPs (18). Fourth, the MR-Steiger method was used to calculate the variance explained by exposure and outcome to avoid reverse causality. Fifth, if an SNP was unavailable in CAD traits, a proxy SNP (*r*^2 ^> 0.8) was used. Finally, we searched for pleiotropic SNPs associated with confounders on the PhenoScanner website (http://www.phenoscanner.medschl.cam.ac.uk/) and used the remaining IVs for further analysis. Supplementary Table S1 lists the characteristics of the included IVs.

### Statistical analyses

For univariable analysis, inverse-variance weighted (IVW) analysis was used as the method for estimation of the causal associations between TIA and CADs. The IVW method assumes that all genetic variants are valid and is most statistically robust when the average pleiotropic effect is zero (19). Considering the potential heterogeneity, we used both the IVW-fixed and IVW-random effect models (20). Cochran's *Q* test was used to evaluate the heterogeneity between the genetic variants. To verify the robustness of the results, we performed a sensitivity analysis with alternative MR models including MR-Egger, weighted median, and MR-PRESSO. The MR-Egger method introduces an intercept term into the Egger regression model and can be used to detect the average horizontal pleiotropy. Besides, this method can produce a valid causal estimate even if all the IVs are invalid (21). The weighted median method can provide a valid estimate when 50% or more SNPs are valid IV (20). Additionally, the MR Pleiotropy Residual Sum and Outlier (MR-PRESSO) test was used for the detection of pleiotropic outliers and providing a causal estimate after the removal of corresponding outliers (22). Finally, the leave-one-out test was used to check whether the causal association was affected by a single SNP (20). The MR Steiger test was performed to estimate the potential reverse causal relationship between TIA and CAD (23). For multivariable MR, the multiplicative IVW random-effect model was used with adjustment for traditional CAD risk factors including smoking, hypertension, and diabetes.

Estimates of the effects of variables on the causal associations between TIA and CAD are presented as odds ratios (ORs) with 95% confidential intervals (CIs). The “TwoSampleMR”, “MVMR”, and “MR-PRESSO” packages in R software, version 4.2.2, were used to conduct this MR analysis.

## Results

### Characteristics of included SNPs

In this study, 18 SNPs were selected after filtering by the significance threshold (*P* < 5 × 10^−6^) and removal of SNPs with LD (*r*^2 ^< 0.01, 10,000 kb). We searched the selected SNPs in the PhenoScanner database to exclude SNPs linked to confounders. One SNP, rs4776884, was excluded due to its association with body fat, hip circumference, and basal metabolic rate. Two SNPs were eliminated from the harmonization of TIA and outcomes (CAA, MI, and IHD) because they were palindromic and had intermediate allele frequencies (rs2461030, rs117382396). Thus, 15 SNPs were finally included as the IVs for TIA. The *F*-statistics for all SNPs were greater than 10. Supplementary Tables S1, S2 show the characteristics of the SNPs.

### Causal association between TIA and CADs

**Figure 2:**
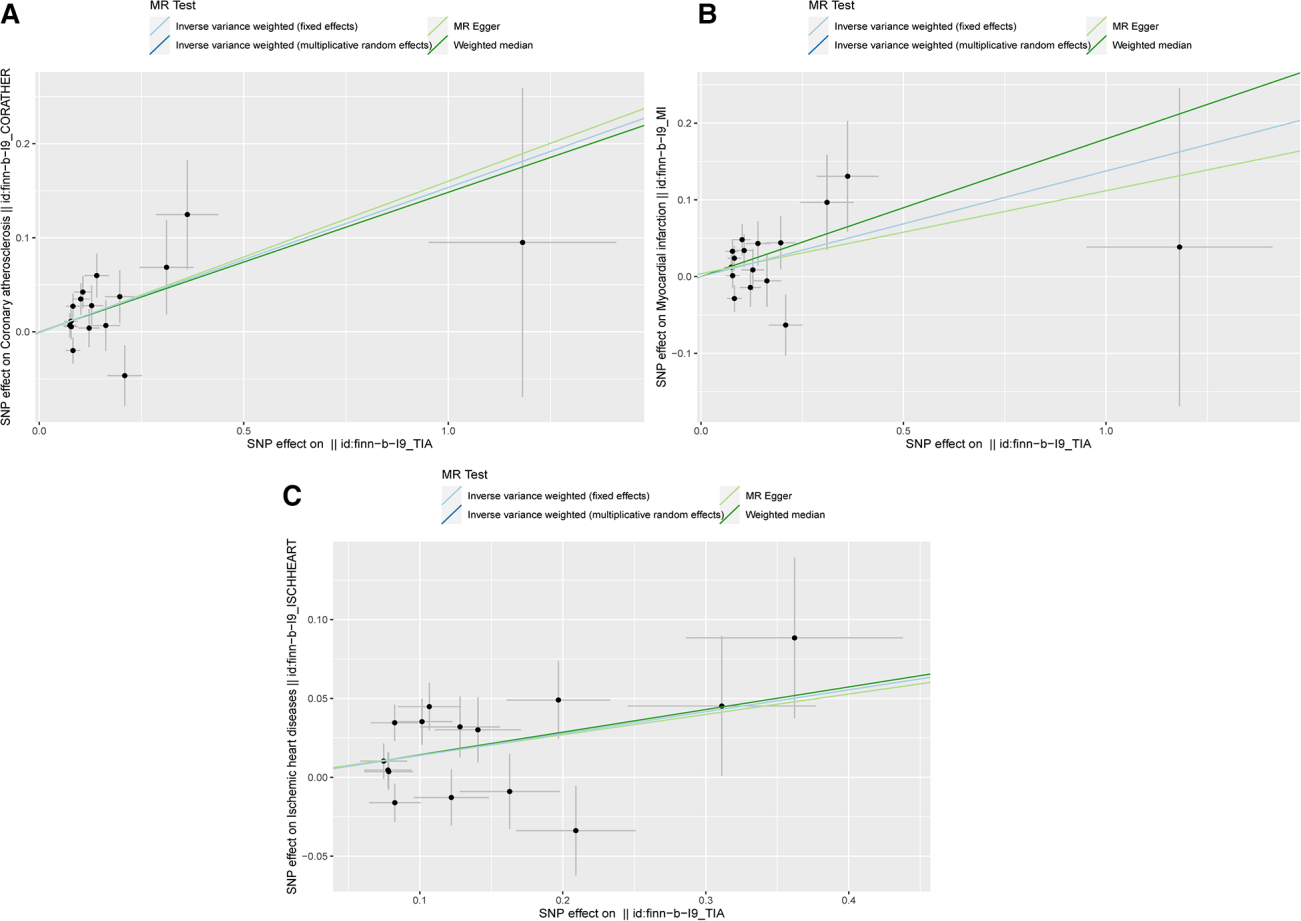
The scatter plots of MR analysis. (**A**) The scatter plot of the causality between TIA and CAA; (**B**) The scatter plot of the causality between TIA and MI; (**C**) The scatter plot of the causality between TIA and IHD.

**Figure 3:**
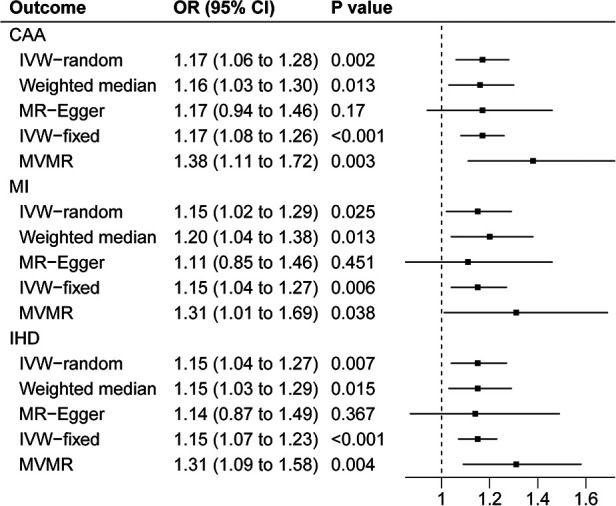
Forest plot of MR analysis.

The scatter plots in Figure 2 showed that the SNP effect on CAA, MI, and IHD increased in correspondence with their effect on TIA. The results of the causal association between TIA and CADs are shown in Figure 3. In both the IVW fixed and random effect models in the univariable MR analysis, genetically predicted TIA was found to increase the risk of CAA, MI, and IHD (OR = 1.17, *P* < 0.05; OR = 1.15, *P* < 0.05; OR = 1.15, *P* < 0.05, respectively). In sensitivity analyses, the association between TIA and CADs remained consistent in the weighted median method (CAA: OR = 1.116, *P* = 0.013; MI: OR = 1.20, *P* = 0.013; IHD: OR = 1.15, *P* = 0.015, respectively). However, the associations were not significant in the MR-Egger analysis (*P* > 0.05). Further multivariable MR analysis adjusted for hypertension, diabetes, and smoking revealed consistent positive estimates for the associations between TIA and CADs (CAA: OR = 1.38, *P* = 0.003; MI: OR = 1.31, *P* = 0.038; IHD: OR = 1.31, *P* = 0.004, respectively).

### Heterogeneity and pleiotropy analyses

The results of the heterogeneity and pleiotropy analyses are shown in Table 2. In the heterogeneity test, Cochran's *Q* statistic showed heterogeneity in the outcome of IHD (*P* < 0.05). Thus, the IVW random-effect model was used, which indicated consistent results. The MR-Egger intercept for all outcomes showed no substantial pleiotropy (intercept *P* > 0.05). The MR-PRESSO global test revealed significant horizontal pleiotropy in the causal association between TIA and IHD but did not detect any significant outliers. There was no evidence of horizontal pleiotropic effects in the associations of TIA and other outcomes. To analyze the effects of single SNPs, the leave-one-out method was used, indicating that the causal association between TIA and CADs was not driven by individual SNPs. The funnel plots and leave-one-out analysis plots are shown in Supplementary Figures S1, S2. The results of the MR Steiger test showed no evidence of reverse causality (Supplementary Table S3).

**Table 2:** Analyses of heterogeneity and pleiotropy.

Outcome	MR-PRESSO	MR-Egger intercept (*P* val.)	Heterogeneity test	
			MR Egger *P* val.	IVW *P* val.
CAA	0.117	−0.001 (0.939)	0.097	0.07
MI	0.09	0.004 (0.816)	0.066	0.09
IHD	0.026	0.004 (0.755)	0.01	0.016

## Discussion

This MR analysis demonstrated the causal relationship between TIA and CAD. The results indicated that the genetic liability to TIA was associated with a higher risk of CAD. The associations remained robust after adjusting for hypertension, diabetes, and smoking in the multivariable MR analysis.

Observational studies demonstrated a relationship between TIA and CAD. In terms of short-term prognosis, CAD was a major cause of hospital readmission within 30 days of an acute stroke or TI, with a prevalence of 17.8% (24). Patients with TIA should also be aware of the long-term risk of developing CAD. A retrospective cohort study focusing on the 5-year outcomes for TIA patients suggested that 53% had at least one cardiometabolic condition (simultaneous coexistence of diabetes mellitus, CAD, heart failure, or atrial fibrillation), and 32% developed CAD (25). Results from the REACH registry of atherothrombosis show that patients with a history of TIA/stroke had a higher rate of cardiovascular events (HR 1.52, 95% CI 1.40–1.65) (26). Furthermore, a previous meta-analysis of 58 studies revealed that 1.67% of individuals with a history of ischemic stroke or TIA are at risk of developing MI (13). Although the risk of severe CAD (such as MI) after TIA is low, asymptomatic CAD is prevalent in patients with TIA. Previous research showed that asymptomatic CAD is common in patients with cerebrovascular disease, with 40% of patients having severe CAD (greater than 70% stenosis) (27). The Predicting Asymptomatic Coronary Artery Disease in Patients With Ischemic Stroke and Transient Ischemic Attack (PRECORIS) study showed that the prevalence of ≥50% asymptomatic CAD is 18% (28). Another large-scale study on Japanese ischemic stroke patients indicated that 23.7% were diagnosed with myocardial ischemia after myocardial scintigraphy (29). The present MR analysis provides new evidence of the causal association between TIA and CADs using large GWAS summary data. As the observational studies indicate that TIA and stroke have similar cardiac complications, the prevention of cardiac events in TIA patients could refer to the integrated care approach to stroke. The importance of antiplatelet and lipid-lowering therapy has been widely discussed, together with the importance of maintaining a healthy lifestyle is also essential (30). Excluding the integrated care approach, regular follow-up of TIA patients could reduce the recurrence of cardiovascular events (31).

For patients with CAD and TIA, atherosclerosis is considered the main pathology. Therefore, common risk factors such as hypertension, diet, smoking, and diabetes mellitus could increase the incidence of CAD in TIA patients (8, 32). However, further explanations for the association between TIA and CAD are lacking. The heart-brain axis, which describes the interactions between cardiovascular illness and the neurological system, could be a potential explanation. Neurological disorders like ischemic stroke produce oxidative stress, which causes a maladaptive increase in sympathetic tone, resulting in arrhythmia or myocardial ischemia (33). Besides, research showed that neuroendocrine changes following TIA may impact the cardiovascular system. The abnormal activation of the hypothalamic–pituitary–adrenal (HPA) axis induced by TIA may cause hypercortisolism, which increases the risk of CAD by affecting both coagulation and lipid metabolism (34). In a case-control study, the data showed that patients with ischemic cerebrovascular disease had reduced levels of high-density lipoprotein (HDL) cholesterol (35). A prospective cohort study including 792 patients who have suffered ischemic stroke/TIA showed that patients with atherogenic dyslipidemia were at higher risk of cardiovascular events (36). Alterations in lipid metabolism have also been linked to endothelial dysfunction and increased coagulation, both of which are risk factors for recurrent vascular disease (37). Other potential mechanisms for an increased risk of CAD following TIA include hypercoagulation and thrombosis. The result of a cross-sectional study showed that patients with previous TIA had higher overall homeostatic and coagulation potentials, together with lower overall fibrinolytic potential (38). Furthermore, a cohort study that included 5,114 patients with cerebrovascular disease also suggested that the risk of coronary events increased linearly with fibrinogen levels (39). These mechanisms require further investigation.

The strength of this study was that it is the first confirmation of a causal relationship between TIA and CAD using two-sample MR analysis. Additionally, we evaluated potential pleiotropy and employed various methods to ensure consistency. Nevertheless, the study has several limitations. First, the results from the different MR methods were inconsistent with those using the IVW method, despite the same overall trends shown by the different estimates. Furthermore, the Cochran *Q* test for the IVW method indicated heterogeneity. This led to the use of the IVW-random effect model was conducted. Second, due to the unavailability of original data, analysis of GWAS data from TIAs of different etiologies was not possible. Third, the subjects of the studies were of European ancestry only, and the results might thus not necessarily be generalized to other ethnicities. Fourth, the MR-PRESSO test showed evidence of pleiotropy in the association between TIA and IHD. Although subsequent multivariable analysis revealed significant estimates, the result should be interpreted with caution. Fifth, the diagnosis of TIA depends on the quality and quantity of information available and the time of assessment and is thus primarily a clinical diagnosis. Inaccurate descriptions from patients and incomplete neurological examinations may result in different diagnoses (28). However, the present MR analysis used summary-level GWAS data of TIA cohorts from the FinnGen project. Potential diagnostic bias in the original research is difficult to adjust. In the future, diagnostic criteria for TIA should be comprehensive and objective to reduce potential bias in observational studies.

## Conclusion

In conclusion, the present study investigated the causal relationship between TIA and CAD using MR analysis. The results showed that TIA could increase the risk of CAD. Further studies are required to verify these conclusions and investigate potential mechanisms.

## Data Availability

The original contributions presented in the study are included in the article/**Supplementary Material**, further inquiries can be directed to the corresponding author.
