# Supplementary material for: Transient ischemic attack and coronary artery disease: a two-sample Mendelian randomization analysis
Source: Front Cardiovasc Med. 2023 Aug 21;10:1192664. doi: 10.3389/fcvm.2023.1192664 (PMC10475993; doi:10.3389/fcvm.2023.1192664)

Transient ischemic attack and coronary artery disease: A two-sample Mendelian randomization analysis

**Supplementary Tables**

**Supplementary Table 1:** Characteristics of genetic instruments associated with transient ischemic attack;

| SNP | chr | A1 | A2 | beta | pos | se | pval | F |
| --- | --- | --- | --- | --- | --- | --- | --- | --- |
| rs4414012 | 1 | T | C | -0.0782 | 4221435 | 0.0171 | 4.79E-06 | 20.91087 |
| rs76139836 | 1 | T | C | -0.1406 | 2.03E+08 | 0.0302 | 3.16E-06 | 21.67235 |
| rs34280651 | 1 | C | T | 0.1015 | 14963316 | 0.0213 | 2.01E-06 | 22.70492 |
| rs2017755 | 5 | T | C | 0.0824 | 1.49E+08 | 0.0167 | 8.24E-07 | 24.3425 |
| rs62394638 | 6 | C | A | -0.0825 | 40609391 | 0.0179 | 4.27E-06 | 21.23987 |
| rs71532312 | 8 | T | C | -0.2091 | 95386247 | 0.0418 | 5.73E-07 | 25.02061 |
| rs2461030* | 8 | C | G | -0.0806 | 80635016 | 0.0163 | 7.03E-07 | 24.44772 |
| rs79982891 | 8 | A | G | -0.1281 | 81922009 | 0.028 | 4.94E-06 | 20.92825 |
| rs79583270 | 8 | T | C | -0.1628 | 73845565 | 0.0349 | 3.05E-06 | 21.7574 |
| rs11528095 | 10 | G | A | -0.0777 | 97479474 | 0.0165 | 2.40E-06 | 22.17289 |
| rs59394373 | 11 | G | C | 0.122 | 33831874 | 0.0261 | 2.91E-06 | 21.84678 |
| rs1022960 | 13 | C | T | 0.0747 | 51724935 | 0.0163 | 4.83E-06 | 20.99987 |
| rs4776884* | 15 | T | C | 0.0741 | 67381340 | 0.0162 | 4.85E-06 | 20.91978 |
| rs2611651 | 15 | T | C | 0.3112 | 91235390 | 0.0658 | 2.27E-06 | 22.36532 |
| rs79582868 | 16 | T | C | -0.1969 | 83362113 | 0.0361 | 5.14E-08 | 29.7447 |
| rs4404139 | 17 | C | T | -0.1066 | 71214587 | 0.0221 | 1.39E-06 | 23.26354 |
| rs117293526 | 22 | G | A | 0.3621 | 23677149 | 0.0759 | 1.85E-06 | 22.75729 |
| rs117382396* | 22 | C | G | 1.1817 | 23778791 | 0.2294 | 2.60E-07 | 26.53181 |

SNP, single nucleotide polymorphism; A1, effect allele; A2, other allele; se, standard error; *: excluded SNP in final analysis

Supplementary Table 2: Genotype-phenotype associations of selected SNPs.

| SNP | Trait | Beta | P | Sample size |
| --- | --- | --- | --- | --- |
| rs4776884 | Sitting height | -0.01191 | 2.45E-10 | 336172 |
| rs4776884 | Weight | -0.01191 | 2.12E-08 | 336227 |
| rs4776884 | Whole body fat mass | -0.01226 | 2.44E-07 | 330762 |
| rs4776884 | Trunk fat mass | -0.01238 | 4.44E-07 | 331093 |
| rs4776884 | Leg fat mass right | -0.0096 | 8.21E-07 | 331293 |
| rs4776884 | Leg fat mass left | -0.00945 | 9.02E-07 | 331275 |
| rs4776884 | Arm fat mass left | -0.01141 | 1.73E-06 | 331164 |
| rs4776884 | Arm fat-free mass left | -0.00734 | 2.11E-06 | 331159 |
| rs4776884 | Arm fat mass right | -0.01119 | 2.71E-06 | 331226 |
| rs4776884 | Basal metabolic rate | -0.00743 | 4.13E-06 | 331307 |
| rs4776884 | Arm predicted mass left | -0.00708 | 4.40E-06 | 331146 |
| rs4776884 | Hip circumference | -0.01076 | 7.95E-06 | 336601 |
| rs76139836 | Cause of death: alcoholic hepatitis | -0.00502 | 1.80E-07 | 7637 |
| rs76139836 | Treatment with sodium warfarin | 0.000634 | 3.51E-06 | 337159 |
| rs11528095 | Platelet distribution width | -0.01709 | 9.23E-06 | 173480 |

Phenotype for other SNPs were not found on PhenoScanner website.

Supplementary table 3. Results of MR_steiger test.

| Outcomes | r2_exp | r2_exp_adj | r2_out | r2_out_adj | Causal direction |
| --- | --- | --- | --- | --- | --- |
| CAA | 3.24 | 3.24 | 0.042 | 0.042 | True |
| MI | 1.844 | 1.844 | 0.041 | 0.041 | True |
| IHD | 3.24 | 3.24 | 0.025 | 0.025 | True |

r2_exp, estimated variance explained in exposure; r2_out, estimated variance explained in outcome; r2_exp_adj, predicted variance explained in exposure accounting for estimated measurement error; r2_out_adj, predicted variance explained in outcome accounting for estimated measurement error.

# Supplementary Figures

**Figure S1:** Funnel plot of MR analysis of transient ischemic attack on coronary artery disease.


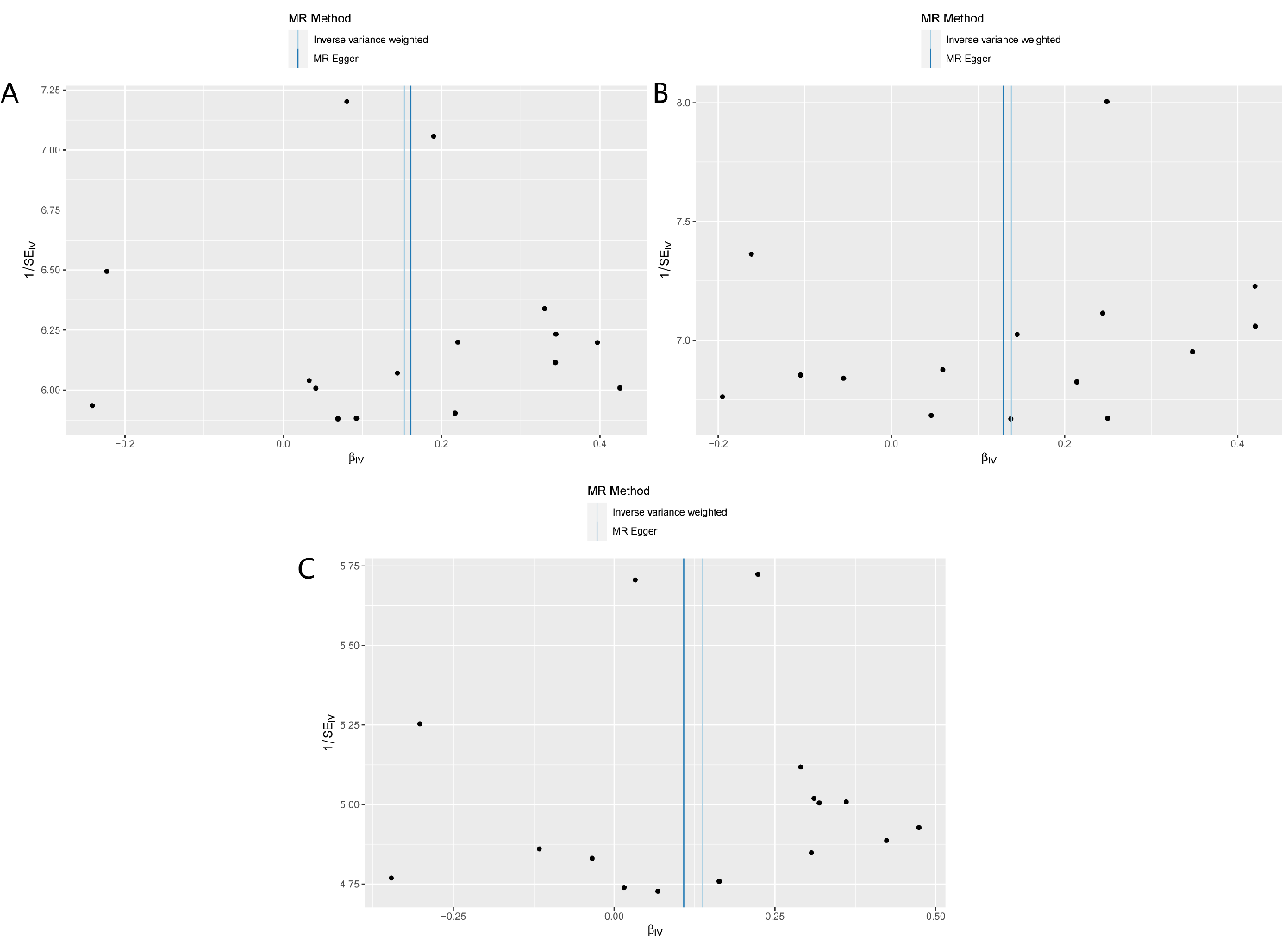


**Figure S2**: Plot from leave-one-out sensitivity analysis for the instrument variable set. The solid lines represent 95% confidence intervals.


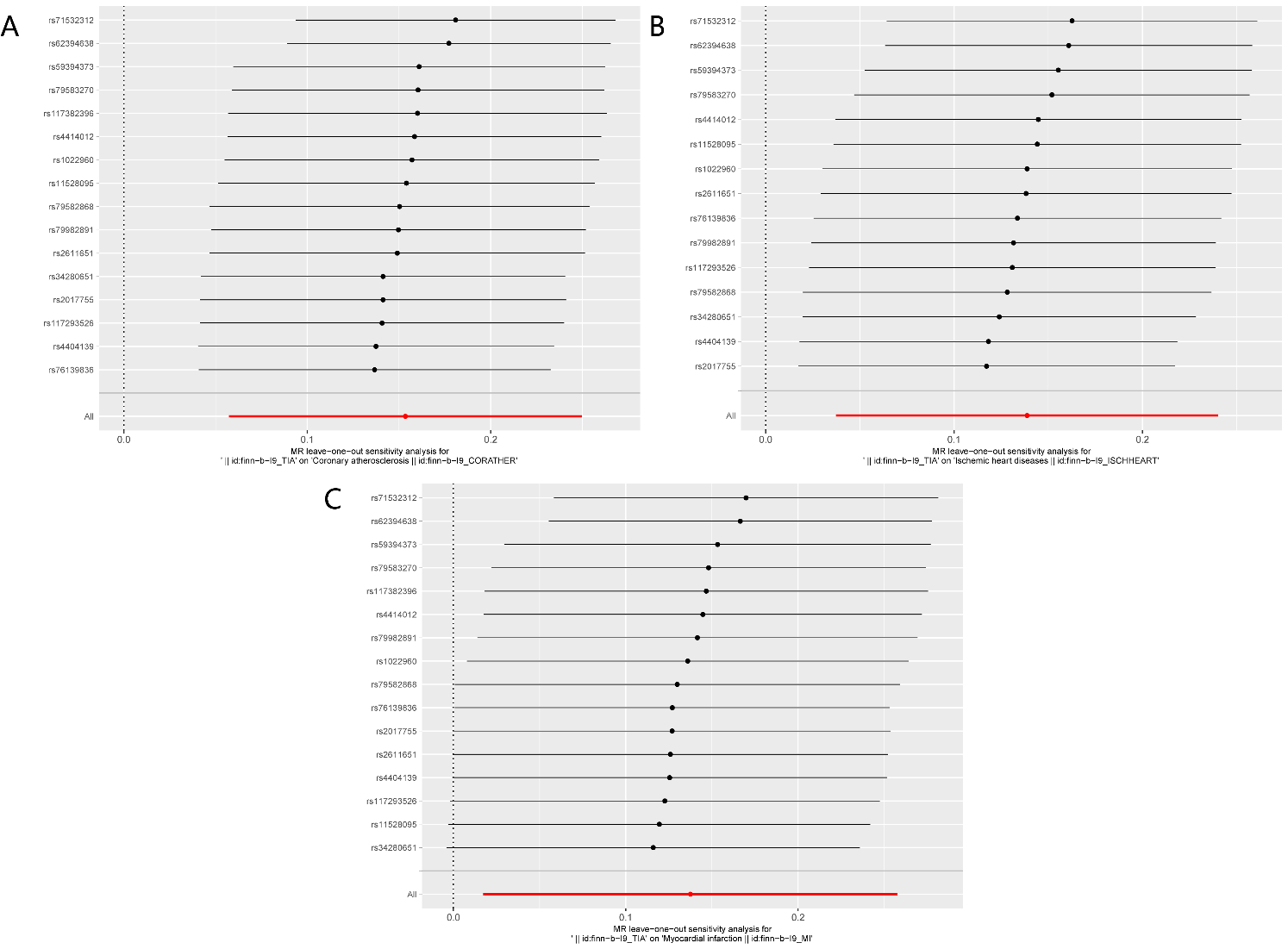

Supplement: Supplementary file 1 [file Datasheet1.docx]
